# Epidemiology and Treatment Trends in the Management of Dupuytren's Disease From 2016 to 2022

**DOI:** 10.7759/cureus.72528

**Published:** 2024-10-28

**Authors:** Nathaniel Pineda, Kassem Ghayyad, Tyler F Beaudoin, David Hirsch, Meysam Fathi, Ibrahim Zeini, Amir R Kachooei

**Affiliations:** 1 Orthopedic Surgery, Drexel University College of Medicine, Philadelphia, USA; 2 Orthopedic Surgery, Rothman Orthopaedics Florida at AdventHealth, Orlando, USA; 3 Orthopedics, University of Central Florida, Orlando, USA; 4 Orthopedics, Rothman Orthopaedics Florida at AdventHealth, Orlando, USA; 5 Orthopedics, Orthopedic Research Center, Mashhad University of Medical Sciences, Mashhad, IRN

**Keywords:** carpal, dupuytren contracture, dupuytren’s disease, hand anatomy, nerve transfer, palmar fasciotomy, scaphoid, tfcc, thumb pollicization, wrist

## Abstract

Background

The United States has been experiencing rapid demographic changes in recent years; therefore, it is crucial to understand how these changes affect the diagnosis and treatment of Dupuytren disease to provide high-quality patient care. This study examines the demographics of Dupuytren disease and its surgical management.

Methods

International Classification of Disease-10 (ICD-10) diagnostic codes were used to query the TriNetX database for all patients diagnosed with Dupuytren disease from 2016 to 2022. Current procedure terminology (CPT) codes were used to stratify the method of surgical interventions for Dupuytren disease and to form patient cohorts.

Results

The incidence of patients diagnosed with Dupuytren disease in the United States from 2016 to 2022 showed a steady annual increase in cases, peaking at 22,711 in 2022 compared to 11,271 in 2016. Most diagnoses occurred in patients aged 65-90 years, accounting for 72% of patients. Dupuytren disease was more prevalent in males (M/F ratio of 1.5/1) and White patients. Notable comorbidities included diabetes in 25% of patients, smoking history in 11% of patients, and hypothyroidism in 17% of patients. Patients with a smoking history had higher rates of surgical intervention, while those with hyperthyroidism had the highest rates of palmar fasciotomies. Males had higher rates of surgical intervention of all types compared to females. Overall, the highest surgical intervention rate was for partial fasciectomy, with single-digit release used in 9.9% of patients, and the lowest for palmar fasciotomy, used only in 1.4% of patients.

Conclusion

This study showed that the rates of Dupuytren disease are rapidly increasing in the United States. Additionally, there are significant differences in the diagnosis and treatment of Dupuytren disease based on patient demographics. Understanding these differences provides an opportunity to explore ways to tailor treatment and allocate resources to best meet patients' needs and improve the quality of care they receive.

## Introduction

Dupuytren's disease is a progressive and irreversible, yet benign, fibroproliferative hand condition characterized by the thickening and tightening of the palmar fascia, which leads to the development of nodules and cords that can cause finger flexion contractures. This condition involves the differentiation of fibroblasts into myofibroblasts, which possess contractile properties and lead to excessive collagen deposition in a disorganized extracellular matrix [1,2]. As a result, progressive contractures of the fingers may occur, most commonly affecting the ulnar side of the hand, although any finger can be involved.

The prevalence of Dupuytren's disease varies geographically and ethnically, with higher rates observed in Northern European patients. It is most commonly seen in older adults, particularly males above the age of 50 [3]. As the condition progresses, it can significantly impair hand function, leading to challenges in performing daily activities and impacting the quality of life. Despite its prevalence, the ideal treatment for Dupuytren's disease is yet to be standardized. Current treatment options range from non-surgical interventions, such as collagenase injections and needle aponeurotomy, to surgical procedures like fasciotomy and fasciectomy [4,5]. These treatments aim to release the contractures and improve hand function. However, the choice of treatment depends on various factors, including the severity of the disease, patient comorbidities, and the potential for recurrence.

Despite the advancements in treatment modalities, the economic burden of treating Dupuytren's disease is substantial, given the costs associated with medical consultations, surgical interventions, postoperative care, work absence, and rehabilitation [6]. As the population ages, the prevalence of this condition is expected to rise, posing significant challenges for healthcare delivery while minimizing increasing healthcare costs. The progressive nature of the disease often necessitates repeated interventions, contributing to substantial healthcare costs and resource utilization [6-8]. Our study aims to analyze the treatment trends and demographic factors associated with Dupuytren's disease in the United States from 2016 to 2022 to better understand its burden on the healthcare system. By examining data from the TriNetX Global Collaborative Network database, a large-scale, deidentified patient database, we seek to identify patterns in treatment utilization, demographic disparities, and potential factors influencing these trends. This information is crucial for healthcare providers and policymakers to allocate resources effectively, improve treatment strategies, and ultimately enhance patient outcomes.

## Materials and methods

A retrospective study was conducted utilizing data from the TriNetX Global Collaborative Network database in November 2023, focusing on patients diagnosed with Dupuytren disease from 2016 to 2022 in the United States. The data in this database were deidentified, exempting it from Institutional Review Board oversight.

Global collaborative network

The study utilized the TriNetX Global Collaborative Network, which provides access to over 400 million deidentified patient records, allowing for comprehensive research and collaboration across medical researchers worldwide. Data within the network includes patient demographics, diagnoses, procedures, laboratory results, and medications. Access to the database does not require prior Institutional Review Board (IRB) approval and is facilitated through collaboration with over 200 community and academic healthcare organizations, along with industry partners globally. The data compiled from this database for the study comprises deidentified patient data from numerous large health systems in the United States through electronic medical record data.

Patient cohort identification

A total of 119,793 patients with Dupuytren disease were identified within the specified timeframe of 2016 to 2022 using the International Classification of Diseases, 10th edition (ICD-10) diagnostic code M72.0, which specifically identifies patients diagnosed with this disease.

Surgical procedure identification

After confirming the diagnosis, the study then identified surgical interventions for Dupuytren disease using the following four Current Procedural Terminology (CPT) codes between 2016 and 2022: (1) 26045: fasciotomy of the palm, (2) 26121: fasciectomy of the palm, (3) 26123: fasciectomy of the palm and single digit, and (4) 26125: fasciectomy of the palm and multiple digits.

Demographic and comorbidity data

Demographic information, including age, sex, ethnicity, and race, as well as comorbidities such as smoking history, type 2 diabetes mellitus (T2DM), hyperthyroidism, and hypothyroidism, was extracted for further analysis. Age was stratified into four quartiles: 0-17, 18-39, 40-64, and 65-90. Sex was categorized as male, female, or unknown. Ethnicity was categorized as Hispanic or Latino, not Hispanic or Latino, and unknown. Race was categorized as White, Black, Asian, Native Hawaiian or other Pacific Islander, American Indian or Alaska Native, and unknown.

## Results

Dupuytren disease

**Figure 1:**
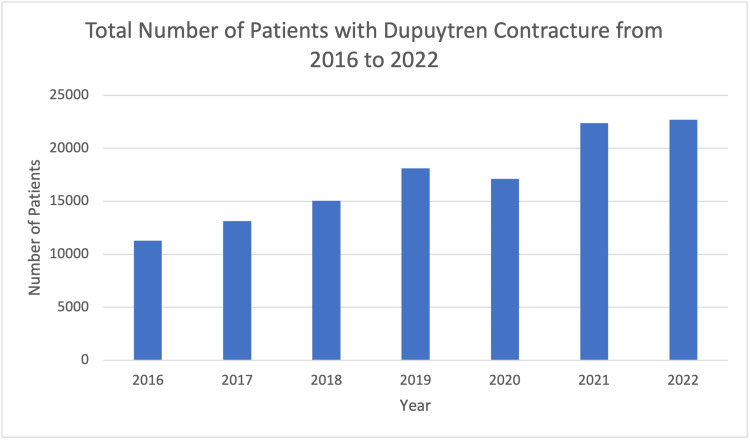
Patients with Dupuytren contracture (2016-2022)

We analyzed data from 119,793 patients diagnosed with Dupuytren disease in the United States from 2016 to 2022. Over this period, the total number of patients diagnosed with Dupuytren disease varied annually, with a peak of 22,711 cases in 2022 and almost half of that in 2016 with 11,271 cases. Although not representative of all patients in the United States diagnosed with Dupuytren disease, data from the TriNetX Global Collaborative Network demonstrated a steady increase in the number of newly diagnosed cases of Dupuytren disease during the study period, more than doubling from 2016 to 2022 (Figure 1).

Demographics of patients with Dupuytren disease

The highest reported diagnosis of Dupuytren disease occurred in patients aged 65 to 90 years, comprising 86,185 patients (72%). The mean age of patients with Dupuytren disease decreased from 72 years in 2016 to 68 years in 2022. The diagnosis was more common in males, with a male-to-female ratio of 1.5:1. This condition was predominantly found in the White population, accounting for 100,875 patients (84%). A smoking history was reported in 11% of patients, while diabetes was present in 25%, hyperthyroidism in 2.1%, and hypothyroidism in 17% of patients (Table 1).

**Table 1:** Incidence rate of Dupuytren disease and patients’ demographics ^1^AI or AN: American Indian or Alaska Native. ^2^NH or OPC: Native Hawaiian or other Pacific Islanders. ^3^Hx: History. Smoking history F17.210. ^4^T2DM: Type 2 diabetes mellitus E11. ^5^Hyperthyroidism E05. ^6^Hypothyroidism E03.9.

	2016	2017	2018	2019	2020	2021	2022	2016-2022
Total	11,271	13,129	15,057	18,122	17,129	22,374	22,711	119,793
*Age*								
Mean age ± SD	72 ± 10.7	71.4 ± 10.9	70.9 ± 10.7	70.3 ± 10.7	69.3 ± 10.7	68.8 ± 10.6	68.2 ± 10.8	
0-17	26	22	14	29	12	15	11	129
18-39	84	128	150	199	204	288	346	1399
40-64	2391	3036	3597	4588	4897	6589	7043	32141
65-90	8770	9943	11296	13306	12016	15482	15372	86185
*Sex*								
Male	6815	7871	8881	10657	10018	12572	13014	69828
Female	3984	4724	5407	6564	6254	8722	8734	44389
Unknown	472	534	769	901	857	1080	963	5576
*Ethnicity*								
Not Hispanic	8898	10275	11577	13974	13509	17702	17561	93496
Unknown	2106	2523	3107	3715	3229	4131	4530	23341
Hispanic	267	331	373	433	391	541	620	2956
*Race*								
White	9517	11110	12562	15121	14425	18825	19315	100875
Unknown	1227	1372	1690	2094	1878	2404	2253	12918
Asian	46	75	81	105	97	140	158	702
Black	266	328	398	470	411	586	542	3001
Other race	181	210	284	283	258	364	380	1960
AI or AN^1^	26	29	39	40	43	44	45	266
NH or OPC^2^	10	10	10	10	17	11	18	86
*Comorbidities*								
Smoking Hx^3^	1374	1510	1700	2010	1821	2195	2193	12803
T2DM^4^	3052	3429	3892	4685	4268	5358	5362	30046
Hyperthyroidism^5^	242	283	321	426	371	464	458	2565
Hypothyroidism^6^	1973	2311	2658	3156	2944	3752	3660	20454

Treatment trends of Dupuytren disease (2016-2022)

Palmar fasciotomy (CPT® code 26045) was performed in 1,622 (1.4%) patients from 2016 to 2022. The procedure was performed more in males aged 40 to 64 years, especially among the Hispanic or Latino and American Indian or Alaska Native race groups (Table 2).

**Table 2:** Rate of palmar fasciotomy ^1^AI or AN: American Indian or Alaska Native. ^2^NH or OPC: Native Hawaiian or other Pacific Islanders. ^3^Hx: History. Smoking history F17.210. ^4^T2DM: Type 2 diabetes mellitus E11. ^5^Hyperthyroidism E05. ^6^Hypothyroidism E03.9. CPT: Current procedure terminology.

CPT® 26045	2016	2017	2018	2019	2020	2021	2022	2016-2022
Total	1.72	1.48	1.38	1.45	1.29	1.17	1.24	1.35
*Age*								
Mean age ± SD								
0-17	0	0	0	0	0	0	0	0
18-39	2.38	2.34	0.67	1.51	0.98	1.04	0.58	1.14
40-64	1.76	1.71	1.72	1.79	1.27	1.26	1.28	1.47
65-90	1.71	1.40	1.28	1.33	1.31	1.13	1.24	1.31
*Sex*								
Male	1.82	1.61	1.45	1.45	1.17	1.08	1.24	1.36
Female	1.03	0.93	0.94	1.16	1.15	1.01	0.96	1.03
Unknown	6.14	4.31	3.64	3.44	3.73	3.43	3.74	3.87
*Ethnicity*								
Not Hispanic	1.55	1.46	1.40	1.37	1.18	1.10	1.24	1.30
Unknown	2.47	1.70	1.35	1.78	1.80	1.43	1.24	1.61
Hispanic	3.75	3.02	2.68	2.31	2.56	1.85	1.61	2.37
*Race*								
White	1.53	1.37	1.32	1.34	1.15	1.06	1.14	1.24
Unknown	3.34	2.33	1.95	2.15	2.40	2.16	2.00	2.27
Asian	0.00	0.00	0.00	9.52	10.31	7.14	6.33	5.70
Black	3.76	3.05	2.51	2.13	2.43	1.71	1.85	2.33
Other race	5.52	4.76	3.52	3.53	3.88	2.75	2.63	3.57
AI or AN^1^	0.00	34.48	0.00	25.00	23.26	22.73	0.00	15.04
NH or OPC^2^	100.00	0.00	0.00	0.00	0.00	0.00	0.00	11.63
*Comorbidities*								
Smoking Hx^3^	1.82	2.12	1.47	1.79	1.70	1.78	1.92	1.80
Diabetes T2DM^4^	1.74	1.17	1.28	1.39	1.27	1.23	1.34	1.33
Hyperthyroidism^5^	4.13	3.53	3.12	2.35	2.70	2.16	2.18	2.73
Hypothyroidism^6^	1.37	1.08	1.20	1.52	1.32	1.39	1.17	1.30

Partial fasciectomy of the palm (CPT® code 26121) was performed at an average rate of 2.2% from 2016 to 2022. The procedure was performed more in males aged 40 to 64 years and American Indian or Alaska Native race group (Table 3).

**Table 3:** Rate of partial fasciectomy of the palm ^1^AI or AN: American Indian or Alaska Native. ^2^NH or OPC: Native Hawaiian or other Pacific Islanders. ^3^Hx: History. Smoking history F17.210. ^4^T2DM: Type 2 diabetes mellitus E11. ^5^Hyperthyroidism E05. ^6^Hypothyroidism E03.9. CPT: Current procedure terminology.

CPT® 26121	2016	2017	2018	2019	2020	2021	2022	2022-2016
Total	2.94	2.64	2.66	2.15	2.18	1.75	1.54	2.16
Age								
Mean Age ± SD								
0-17	0.00	0.00	0.00	0.00	0.00	0.00	0.00	0.00
18-39	3.57	2.34	1.33	3.02	1.47	0.35	1.16	1.57
40-64	3.97	3.69	3.31	2.79	2.70	2.05	2.02	2.69
65-90	2.66	2.33	2.48	1.92	1.98	1.65	1.32	1.97
Sex								
Male	3.07	2.93	2.85	2.38	2.35	1.87	1.67	2.34
Female	2.74	2.14	2.42	1.89	2.01	1.72	1.32	1.93
Unknown	2.75	2.81	2.21	1.33	1.40	0.93	1.77	1.72
Ethnicity								
Not Hispanic	1.75	2.62	2.74	2.23	2.18	1.77	1.52	2.06
Unknown	3.04	2.66	2.35	1.83	2.07	1.38	1.43	1.98
Hispanic	4.12	3.32	2.95	2.54	2.81	3.70	2.74	0.00
Race								
White	3.09	2.61	2.75	2.17	2.18	1.81	1.49	2.18
Unknown	1.96	2.11	1.95	1.34	1.81	1.08	1.60	1.63
Asian	0.00	13.33	12.35	9.52	10.31	7.14	6.33	8.55
Black	3.76	4.88	3.77	3.19	2.92	2.56	1.85	3.10
Other race	5.52	4.76	3.52	4.24	3.88	2.75	2.63	3.67
AI or AN^1^	38.46	34.48	25.64	25.00	23.26	22.73	22.22	26.32
NH or OPC^2^	0.00	0.00	0.00	0.00	0.00	0.00	0.00	0.00
Comorbidities								
Smoking Hx^3^	4.73	3.91	3.53	3.13	2.47	2.00	1.87	2.94
Diabetes T2DM^4^	3.51	2.77	3.01	2.56	2.09	1.87	1.57	2.37
Hyperthyroidism^5^	4.55	3.53	4.05	2.35	2.70	2.16	2.18	2.88
Hypothyroidism^6^	2.94	2.64	3.01	2.03	2.17	1.92	1.28	2.18

Partial fasciectomy of the palm with the release of the single digit (CPT® code 26123) was performed at an average rate of 9.9% from 2016 to 2022. The procedure was performed more in males aged 40 to 64 years and among Native Hawaiians or other Pacific Islanders race groups. A higher rate of smoking and type II diabetes were reported in patients who underwent partial fasciectomy of the palm with the release of the single digit (Table 4).

**Table 4:** Rate of partial fasciectomy of the palm with the release of the single digit ^1^AI or AN: American Indian or Alaska Native. ^2^NH or OPC: Native Hawaiian or other Pacific Islanders. ^3^Hx: History. Smoking history F17.210. ^4^T2DM: Type 2 diabetes mellitus E11. ^5^Hyperthyroidism E05. ^6^Hypothyroidism E03.9. CPT: Current procedure terminology.

CPT 26123	2016	2017	2018	2019	2020	2021	2022	2016-2022
Total	11.45	11.42	11.64	10.68	9.89	8.42	7.68	9.85
Age								
Mean Age ± SD								
0-17	0.00	0.00	0.00	0.00	0.00	6.67	9.09	1.55
18-39	11.90	7.81	10.00	6.03	8.33	6.60	6.36	7.51
40-64	12.25	12.19	12.20	11.57	10.72	9.06	8.06	10.34
65-90	11.30	11.28	11.49	10.47	9.59	8.19	7.51	9.72
Sex								
Male	13.54	13.53	13.52	12.51	11.58	10.24	9.24	11.70
Female	8.01	8.04	8.32	7.43	7.40	5.65	5.20	6.86
Unknown	10.38	10.11	13.13	12.76	8.28	9.63	9.24	10.46
Ethnicity								
Not Hispanic	11.55	11.65	12.05	10.90	10.32	8.47	7.87	10.07
Unknown	11.16	10.62	10.49	7.51	8.39	7.96	6.89	8.65
Hispanic	10.49	10.27	9.12	7.85	7.42	10.54	8.23	0.00
Race								
White	11.77	11.61	11.84	10.77	10.38	8.62	7.72	10.05
Unknown	10.43	10.50	11.12	10.55	7.51	7.90	7.90	9.21
Asian	21.74	13.33	12.35	9.52	10.31	7.14	6.33	9.97
Black	8.65	12.50	12.31	11.70	8.03	7.34	7.20	9.43
Other race	6.63	8.10	8.10	7.77	6.20	5.77	5.79	6.79
AI or AN^1^	38.46	34.48	25.64	25.00	23.26	22.73	22.22	26.32
NH or OPC^2^	100.00	0.00	0.00	100.00	58.82	0.00	0.00	34.88
Comorbidities								
Smoking Hx^3^	13.90	13.51	13.06	13.33	12.41	11.07	9.76	12.25
Diabetes T2DM^4^	10.55	10.00	9.76	9.41	8.58	7.88	7.16	8.85
Hyperthyroidism^5^	12.40	9.19	10.90	7.04	5.93	5.82	5.68	7.64
Hypothyroidism^6^	9.93	9.65	10.12	8.68	8.83	6.85	6.67	8.42

Partial fasciectomy of the palm with the release of the multiple digits (CPT® code 26125) was performed at an average rate of 4.2% from 2016 to 2022. The procedure was performed more in males aged 18 to 39 years and among non-Hispanic/Latino and American Indian or Alaska Native race groups. A higher rate of smoking and type II diabetes were reported in patients who underwent partial fasciectomy of the palm with the release of multiple digits (Table 5).

**Table 5:** Rate of partial fasciectomy of the palm with the release of the multiple digits ^1^AI or AN: American Indian or Alaska Native. ^2^NH or OPC: Native Hawaiian or other Pacific Islanders. ^3^Hx: History. Smoking history F17.210. CPT: Current procedure terminology.

CPT 26125	2016	2017	2018	2019	2020	2021	2022	2016-2022
Total	5.79	5.26	5.01	4.59	4.02	3.31	2.82	4.17
*Age*								
Mean age ± SD								
0-17	0.00	0.00	0.00	0.00	0.00	0.00	0.00	0.00
18-39	11.90	7.81	6.67	5.03	4.90	3.47	2.89	5.00
40-64	5.77	5.60	5.75	5.25	4.43	3.70	2.91	4.42
65-90	5.85	5.22	4.82	4.42	3.91	3.16	2.78	4.12
*Sex*								
Male	6.96	6.25	6.22	5.68	4.77	4.14	3.57	5.14
Female	3.87	3.64	3.03	2.65	2.78	1.93	1.56	2.57
Unknown	5.30	5.06	4.94	5.77	4.32	4.81	4.15	4.86
*Ethnicity*								
Not Hispanic	5.95	5.43	5.27	4.84	4.18	3.33	2.82	4.30
Unknown	5.32	5.15	3.86	3.82	3.47	3.10	2.65	3.70
Hispanic	4.49	3.93	3.75	3.00	3.07	4.44	3.87	0.00
*Race*								
White	5.88	5.26	5.10	4.61	4.15	3.29	2.78	4.20
Unknown	5.62	5.61	4.79	4.78	3.78	3.95	3.51	4.43
Asian	21.74	13.33	12.35	9.52	10.31	7.14	6.33	9.97
Black	5.64	5.79	4.02	4.26	2.68	2.39	2.03	3.53
Other race	5.52	4.76	4.58	3.53	3.88	2.75	2.63	3.72
AI or AN^1^	38.46	34.48	25.64	25.00	0.00	22.73	22.22	22.56
NH or OPC^2^	0.00	0.00	0.00	0.00	0.00	0.00	0.00	0.00
*Comorbidities*								
Smoking Hx^3^	6.55	6.16	6.24	6.07	5.71	4.74	3.33	5.40

**Figure 2:**
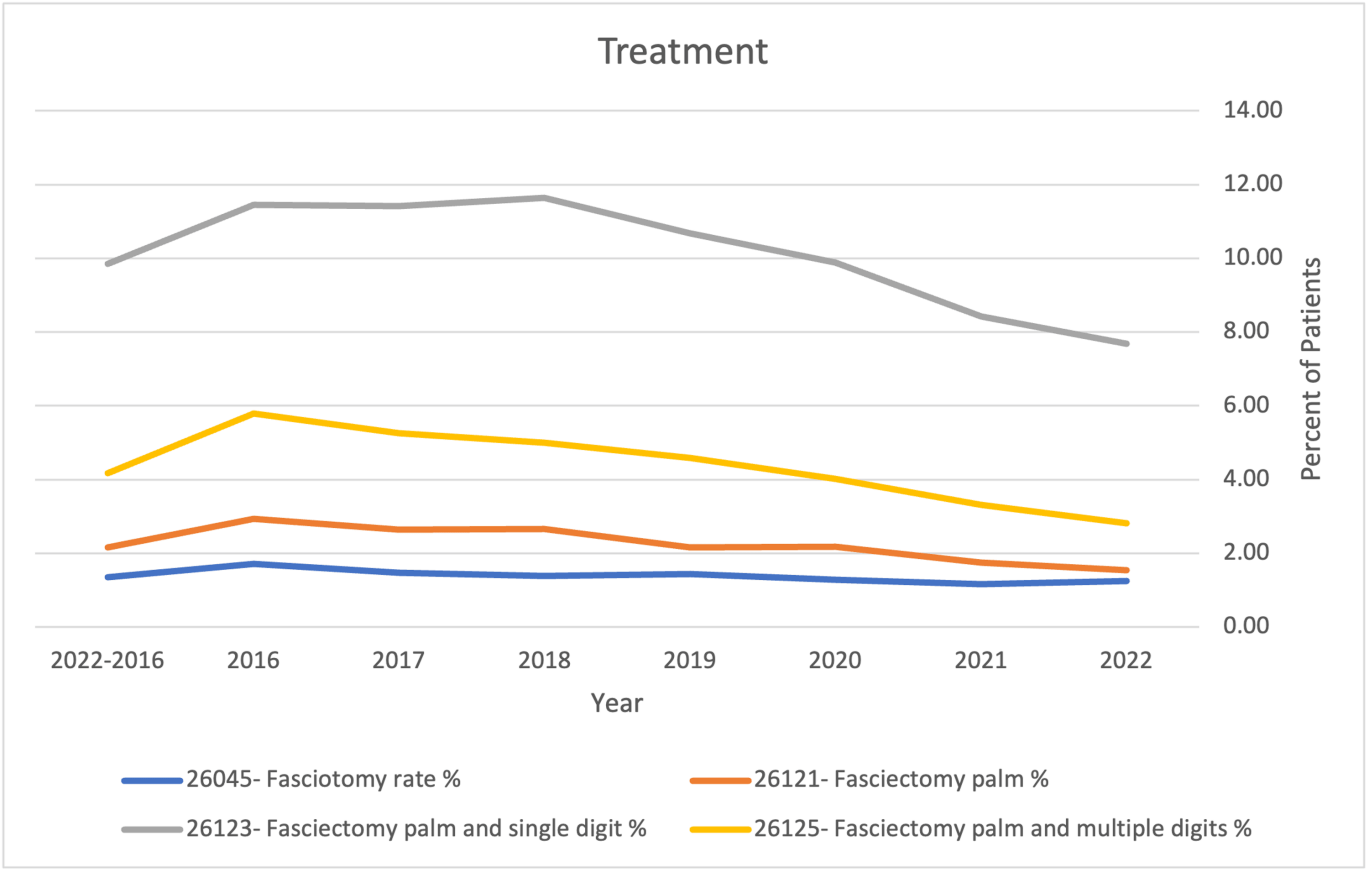
Treatment rates with varying forms of fasciotomies

Overall partial fasciectomy of the palm with release of the single digit had the highest average rate of 9.9% from 2016 to 2022, with palmar fasciotomy having the lowest average rate of 1.4% from the same year range (Table 6 and Figure 2).

**Table 6:** Treatment rates of Dupuytren contracture with varying forms of fasciotomies

Year	2016	2017	2018	2019	2020	2021	2022	2016-2022
Fasciotomy rate %	1.7	1.5	1.4	1.5	1.3	1.2	1.2	1.4
Fasciectomy palm %	2.9	2.6	2.7	2.2	2.2	1.8	1.5	2.2
Fasciectomy palm and single digit %	11	11	12	11	9.9	8.4	7.7	9.9
Fasciectomy palm and multiple digits %	5.8	5.3	5.0	4.6	4.0	3.3	2.8	4.2

Overall, the utilization of these treatments varied across age groups, with patients in the middle age range (40-64) consistently exhibiting the highest rates across all procedures for Dupuytren's disease (Table 7).

**Table 7:** Dupuytren’s contracture treatments among differing age groups

Treatment	Age group	2016	2017	2018	2019	2020	2021	2022	2016-2022
Fasciotomy	0-17	0	0	0	0	0	0	0	0
	18-39	2.38	2.34	0.67	1.51	0.98	1.04	0.58	1.14
	40-64	1.76	1.71	1.72	1.79	1.27	1.26	1.28	1.47
	65-90	1.71	1.40	1.28	1.33	1.31	1.13	1.24	1.31
Fasciectomy palm	Age group	2016	2017	2018	2019	2020	2021	2022	2016-2022
	0-17	0.00	0.00	0.00	0.00	0.00	0.00	0.00	0.00
	18-39	3.57	2.34	1.33	3.02	1.47	0.35	1.16	1.57
	40-64	3.97	3.69	3.31	2.79	2.70	2.05	2.02	2.69
	65-90	2.66	2.33	2.48	1.92	1.98	1.65	1.32	1.97
Fasciectomy palm and single digit	Age group	2016	2017	2018	2019	2020	2021	2022	2016-2022
	0-17	0.00	0.00	0.00	0.00	0.00	6.67	9.09	1.55
	18-39	11.90	7.81	10.00	6.03	8.33	6.60	6.36	7.51
	40-64	12.25	12.19	12.20	11.57	10.72	9.06	8.06	10.34
	65-90	11.30	11.28	11.49	10.47	9.59	8.19	7.51	9.72
Fasciectomy palm and multiple digits	Age group	2016	2017	2018	2019	2020	2021	2022	2016-2022
	0-17	0.00	0.00	0.00	0.00	0.00	0.00	0.00	0.00
	18-39	11.90	7.81	6.67	5.03	4.90	3.47	2.89	5.00
	40-64	5.77	5.60	5.75	5.25	4.43	3.70	2.91	4.42
	65-90	5.85	5.22	4.82	4.42	3.91	3.16	2.78	4.12

Additionally, of the various comorbidities analyzed, patients with a smoking history exhibited a higher rate of undergoing surgical care for their Dupuytren’s disease in comparison to other comorbidities, except palmar fasciotomies. Patients with hyperthyroidism exhibited the highest rates of palmar fasciotomies (Table 8).

**Table 8:** Dupuytren’s contracture treatments among differing comorbidities Hx: History; T2DM: Type 2 diabetes mellitus.

Treatment	Comorbidities	2016	2017	2018	2019	2020	2021	2022	2016-2022
Fasciotomy	Smoking Hx	1.82	2.12	1.47	1.79	1.70	1.78	1.92	1.80
	T2DM	1.74	1.17	1.28	1.39	1.27	1.23	1.34	1.33
	Hyperthyroidism	4.13	3.53	3.12	2.35	2.70	2.16	2.18	2.73
	Hypothyroidism	1.37	1.08	1.20	1.52	1.32	1.39	1.17	1.30
Fasciectomy palm	Comorbidities	2016	2017	2018	2019	2020	2021	2022	2016-2022
	Smoking Hx	4.73	3.91	3.53	3.13	2.47	2.00	1.87	2.94
	T2DM	3.51	2.77	3.01	2.56	2.09	1.87	1.57	2.37
	Hyperthyroidism	4.55	3.53	4.05	2.35	2.70	2.16	2.18	2.88
	Hypothyroidism	2.94	2.64	3.01	2.03	2.17	1.92	1.28	2.18
Fasciectomy palm and single digit	Comorbidities	2016	2017	2018	2019	2020	2021	2022	2016-2022
	Smoking Hx	13.90	13.51	13.06	13.33	12.41	11.07	9.76	12.25
	T2DM	10.55	10.00	9.76	9.41	8.58	7.88	7.16	8.85
	Hyperthyroidism	12.40	9.19	10.90	7.04	5.93	5.82	5.68	7.64
	Hypothyroidism	9.93	9.65	10.12	8.68	8.83	6.85	6.67	8.42
Fasciectomy palm and multiple digits	Comorbidities	2016	2017	2018	2019	2020	2021	2022	2016-2022
	Smoking Hx	6.55	6.16	6.24	6.07	5.71	4.74	3.33	5.40
	T2DM	5.21	4.70	3.88	4.31	3.54	3.32	2.80	3.83
	Hyperthyroidism	6.20	3.89	4.05	2.35	2.70	2.16	2.18	3.08
	Hypothyroidism	5.07	4.98	4.33	4.06	3.60	2.67	2.35	3.67

## Discussion

Our study aimed to analyze the prevalence and treatment trends of Dupuytren's disease in the United States from 2016 to 2022 and to understand its burden on the healthcare system. Our analysis identified 119,793 patients diagnosed with Dupuytren's disease during this period. We observed a steady increase in the number of diagnosed cases each year, with a peak in 2022. The steady increase in the number of diagnosed cases, which more than doubled from 11,271 in 2016 to 22,711 in 2022, underscores the growing recognition and possibly the rising incidence of Dupuytren disease in the United States. This trend may reflect an increased awareness among healthcare providers and patients, improved diagnostic capabilities, or an actual rise in disease prevalence [6,8,9]. The majority of patients were older adults, predominantly males, and of the White race, and notable associations with comorbidities such as diabetes and smoking were found [10-12].

The comorbidity analysis revealed notable associations with smoking, type 2 diabetes mellitus (T2DM), hyperthyroidism, and hypothyroidism [11]. These findings are significant as they corroborate the established risk factors for Dupuytren disease, particularly the strong association with diabetes and smoking, both of which are known to influence the disease's pathophysiology [1,3,6]. The association between diabetes and smoking corroborates previous findings, as these factors influence the disease's pathophysiology through mechanisms involving fibroblast proliferation and altered wound healing [1,9].

The study identified four primary surgical procedures used to manage Dupuytren disease: fasciotomy, partial fasciectomy of the palm, partial fasciectomy with the release of a single digit, and partial fasciectomy with the release of multiple digits [10]. The rates of these procedures varied, with partial fasciectomy with the release of a single digit being the most common at 9.9% of patients, followed by partial fasciectomy with the release of multiple digits in 4.2% of patients, partial fasciectomy of the palm with 2.2% of patients, and fasciotomy in 1.4% of patients. Interestingly, surgical interventions were more common in males and varied significantly across age and racial groups. For instance, palmar fasciotomy had a higher incidence in Hispanic or Latino patients at 2.4% and those identifying as American Indian or Alaska Native at 15%. Similarly, partial fasciectomy procedures were notably higher in American Indian or Alaska Native patients, with a striking 26% for partial fasciectomy of the palm. These disparities suggest potential differences in early versus late presentation, disease severity at the time of presentation, healthcare access, or treatment preferences among different racial groups [10].

The higher prevalence of Dupuytren's disease among White patients can be attributed to genetic predisposition, as the condition is more common in individuals of Northern European descent. The male predominance (M/F ratio of 1.5:1) observed in this study is consistent with existing literature, which reports a higher prevalence of Dupuytren disease in males [4,7,9,13]. It is also important to note that other studies have found a higher male-to-female ratio of Dupuytren's disease, with some reporting rates between 3:1 and 9:1 [7]. This may be due to the study population being comprised mainly of patients in the 65-90 age group, which has been shown to have a male-to-female ratio of Dupuytren's disease closer to 1:1. This could explain why the overall ratio is lower thanthat found in other studies [7,9]. The demographic analysis highlights that Dupuytren disease predominantly affects older adults, with the highest incidence in patients aged 65-90 years. This finding aligns with the known accumulative age-related nature of the disease [9,13]. The mean age of patients decreased slightly from 72 years in 2016 to 68 years in 2022, suggesting that younger individuals are increasingly being diagnosed, which could be attributed to earlier detection and intervention. The majority of patients were White at 84% of all patients, reflecting the known racial predisposition toward this condition. The higher rates of surgical interventions among specific racial groups, particularly American Indian or Alaska Native patients, may reflect more severe disease at the time of presentation, late presentation, or differing healthcare access, cultural differences, and treatment preferences [10,11]. van Rijssen et al. highlighted that patients in certain demographic groups may experience more severe forms of the disease, necessitating more frequent surgical intervention [8].

The increased rate of surgery in the 40-65 age group may reflect the higher functional demands, earlier onset, and rapidly progressive condition causing more disabling contractures in this patient cohort. Younger individuals in this age group are more likely to seek surgical treatment to maintain hand functionality and quality of life [5].

Interestingly, some cases were identified in the adolescent age group, as well as higher-than-expected rates of Dupuytren's disease in the Black patient group. The higher rates in both groups may be attributed to the data being sourced from large health centers in the United States, which may encounter more abnormal cases of the condition than expected in the other areas of the country. The cases observed in the adolescent population may be explained by underlying genetic factors in these patients receiving care at a large medical center, as this age group is rarely affected by the condition.

The observed decline in surgical interventions over recent years may be due to several factors. Besides an improved diagnosis and recording, advances in non-surgical treatments that can be performed in the office, such as collagenase injections and needle aponeurotomy, have provided effective alternatives to surgery, reducing the overall need for invasive procedures [4]. The use of collagenase injections may explain the decreased rates of surgery in the 65-90 age group, as they may be more likely to pursue non-surgical alternatives [14,15]. Economic factors and the affordability of surgical procedures may also play a role in the observed trends. Additionally, financial constraints and healthcare access disparities could also contribute to the observed trends.

Our findings are consistent with existing literature regarding the demographic and comorbidity profiles associated with Dupuytren disease [11]. Previous studies have similarly noted the male predominance and the association with diabetes and smoking [5,6,9,11]. However, our study adds to the understanding by providing a large-scale analysis of treatment trends and demographic variations, highlighting the disparities in surgical intervention rates across different racial and gender groups. For instance, Gudmundsson et. al. corroborates the higher incidence in older males and underscores the genetic and environmental risk factors contributing to the disease [14]. This underscores the need for tailored approaches in managing Dupuytren disease, considering the varying disease severity and healthcare access among diverse populations [11].

While this study provides valuable insights into the epidemiology and treatment trends of Dupuytren disease, several limitations must be acknowledged. The retrospective nature of the study and reliance on ICD-10 and CPT codes for identifying cases and procedures may introduce coding inaccuracies as variations in coding practices, and healthcare access across different regions could influence the observed trends. Additionally, the deidentified nature of the data limits the ability to assess individual patient outcomes and long-term follow-up. Additionally, our dataset may not capture all potential confounding factors influencing the trends observed. Future research should aim to explore the underlying reasons for demographic and racial disparities in disease presentation and treatment. Moreover, investigating the impact of lifestyle modifications and medical therapies on disease progression could offer alternative management strategies for patients with Dupuytren disease [16].

## Conclusions

This research highlights the increasing occurrence of Dupuytren's disease in the United States. It emphasizes the importance of early diagnosis, patient education, and strategic healthcare planning for effective disease management. The rising rates of Dupuytren's disease are associated with the growing elderly population in the United States and the increasing number of related health conditions. Additionally, patient demographic factors may influence the choice of surgical management. Improving our understanding of Dupuytren's disease and its management is essential to provide better support for affected individuals and reduce its impact on the healthcare system.
